# Intra-session reliability of electromyographic measurements in flywheel squats

**DOI:** 10.1371/journal.pone.0243090

**Published:** 2020-12-03

**Authors:** Darjan Spudić, Darjan Smajla, Nejc Šarabon

**Affiliations:** 1 Faculty of Sport, University of Ljubljana, Ljubljana, Slovenia; 2 Faculty of Health Sciences, University of Primorska, Izola, Slovenia; 3 InnoRenew CoE, Izola, Slovenia; 4 S2P, Science to Practice, Ltd., Laboratory for Motor Control and Motor Behaviour, Ljubljana, Slovenia; 5 University of Primorska, Andrej Marušič Institute, Koper, Slovenia; University of Belgrade, SERBIA

## Abstract

Although the popularity of flywheel (FW) devices in sports research is increasing, to date, no study has been designed to test the reliability of electromyographic (EMG) variables during FW squats as a basic lower-body FW resistance exercise. At the primary level, our study was conducted to determine the minimum number of the consecutive flywheel (FW) squat repetitions that need to be averaged in a single set to obtain excellent reliability of peak, mean and three position-specific EMG variables. At the secondary level, comprehensive analysis for peak and mean EMG variables was done. Intra-set reliability was investigated using the minimum number of repetitions determined from the primary level of the study. Twenty-six participants performed five sets of seven squats with three FW loads (0.05, 0.125, 0.225 kg∙m^2^). EMG signals were collected from eight leg muscles. By averaging twelve consecutive repetitions, we obtained ICC_2.k_ > 0.95 for mean and peak EMG_RMS_ regardless of the muscle, load or phase of the squat (concentric vs. eccentric). Due to the heterogeneity of the results at the primary level, position-specific variables were excluded from the inter-set reliability analysis at the secondary level. Trustworthy mean and peak EMG variables from the primary level showed good to excellent inter-set reliability. We suggest averaging twelve consecutive squat repetitions to achieve good to excellent intra-session reliability of EMG variables. By following the proposed protocol, activation of leg muscles can be confidently studied in intra-session repeated-measures study designs.

## Introduction

Despite the increasing popularity of flywheel (FW) devices, especially in the fields of research, sports and health care, only a several studies have assessed electromyographic (EMG) muscle activation during FW loading conditions [1–10]. Lower EMG activity in the eccentric—compared to concentric—phase of the contraction is obvious for the exercises with equal gravity-based load (i.e. weight-stack or barbell) [11,12]. In contrast, studies using FW load have indicated greater muscle activation during the eccentric phase compared to gravity-based exercises in both open [1] and closed [8] kinetic chain exercises. Most recently Alkner & Bring (2019) [9] measured higher mean EMG activation during the eccentric phase of the contraction when comparing the FW leg press to a following gravity-based resistance (GB) exercises: barbell front squat, weight stack leg press and weight stack knee extension. One of the shortcomings of the recent studies comparing EMG muscle activity between FW and GB resistance exercises was the relativization of load selection (FW vs. weights) and the tempo of the exercise being executed (FW all-out vs. fluent concentric). In this manner, it can also be speculated that performing such GB exercises required a more controlled approach compared to the all-out effort from the first repetition on, applicable in the FW devices. Most of the FW resistance protocols were, therefore, power-oriented and were targeting improvements in neuromuscular activation. In contrast, for the GB resistance exercises, load was determined by the maximum number of repetitions performed with fluent concentric repetitions, meaning that it was submaximal during most of the set repetitions [9]. The variable tempo of the exercise execution using FW resistance, which is oriented towards high power outputs, significantly influences the rate of force development, resulting in burst-like muscle activation patterns that potentially decrease the reliability of measurements [13]. Therefore, the reliability of the EMG variables using FW resistance should be questioned.

Due to stochastic nature of an EMG signal [14], in order to obtain representative insight into EMG activation, the average of consecutive repetitions should be considered. To date, there has been a lack of consensus across studies about the representative number of repetitions and muscles analysed during FW leg press movement patterns. To our knowledge, previous studies used signals from three [9] to ten [4] consecutive repetitions, which were post-hoc averaged. In contrast, an average of five sets of 10 repetitions during the FW squat [8] were used in comparing quadriceps muscle activity between FW and GB resistance. Signals were averaged from the following muscles: m. vastus medialis (*vm*) [4,8–10], m. vastus lateralis (*vl*) [4,8–10], m. rectus femoris (*rf*) [8,9], m. gastrochnemius medialis (*m*.*gas*) and lateralis (l.*gas*) [4]. To date, only one study [4] reported between-participant (n = 17) reliability of mean *vl*, *vm*, *m*.*gas* and *l*.*gas* muscle activation for the concentric and eccentric phase of the squat using intraclass correlation coefficient (ICC) and within-participant coefficient of variation (CV). Reliability was highest for *vm* (ICC = 0.95, CV = 9.9%) and lowest for *l*.*gas* (ICC = 0.22, CV = 17.4%) muscles. Additionally, only Alkner & Bring (2019) [9] analysed position-specific EMG variables during FW leg press movement pattern. EMG activity during the concentric and eccentric actions were averaged over position-based—10° knee angle width—intervals from 85° to 155° knee extension joint angles.

Altogether, questions concerning the reproducibility of EMG variables during FW squats, remain open. To reliably follow training adaptations and related underlying mechanisms in future research, intra-session reliability concerning leg muscles at different FW loading conditions should be assessed. Although the popularity of FW devices in sports research is increasing, no study to date has been specifically designed to test the reliability of EMG variables during FW squats. Consequently, the primary level of our study was conducted to determine the minimum number of consecutive repetitions that need to be averaged to obtain reliable intra-session measures of EMG outcome variables. At the secondary level, the inter-set reliability was investigated using trustworthy EMG variables determined in the primary level. Using three different FW load conditions and signals from eight leg muscles, we hypothesized that averaging a higher number of consecutive repetitions improves the reliability of the selected EMG variables. Three chosen loading conditions (0.05, 0.125, and 0.225 kg∙m2) represent very fast, medium, and slow velocity squat movements, therefore EMG acquisition was covered during equidistantly different training conditions, which are representative of strength, power, or speed regimens. Furthermore, the trustworthy variables from the primary level were expected to provide us with good to excellent inter-set reliability at the secondary level. The results are proposed to contribute to the standardization of the methodology for assessing leg muscle EMG measurements using FW squats.

## Materials and methods

### Participants

Twenty-six physically active volunteers participated in the study—for details see Table 1. The inclusion criterion was strength-training experience (strength exercises at least two times per week in the last five years). The exclusion criteria were: knee injuries, chronic diseases, history of lower back pain or acute injuries in the past 6 months. The study was approved by the National Medical Ethics Committee (no. 0120-690/2017/8) and adhered to the tenets of the Oviedo Convention and Declaration of Helsinki. The individual in this manuscript has given written informed consent (as outlined in PLOS consent form) to publish these case details. Participants were informed about the testing procedures prior to signing an informed consent. They were instructed to avoid any strenuous exercise at least two days prior to the testing session.

**Table 1:** Main characteristics of the participants.

	N	Age (years)	Mass (kg)	Height (m)	Body mass index (kg/m^2^)	Training history (years)
Male	12	25.8 ± 4.4	76.4 ± 8.1	177.6 ± 5.1	25.8 ± 4.4	12.3 ± 3.2
Female	14	24.3 ± 3.3	63.1 ± 7.1	165.3 ± 5.0	24.3 ± 3.3	10.8 ± 2.5
All	26	24.9 ± 3.9	69.3 ± 10.0	171.2 ± 7.8	23.6 ± 2.5	11.4 ± 2.9

*Note*: N, number of subjects; All, male and female; data are presented as means ± standard deviations.

### Experimental design

A repeated-measures design was used to assess (a) the reliability of the EMG outcome variables depending on the number of averaged repetitions and (b) inter-set reliability for each FW load.

### Testing procedures

**Fig 1:**
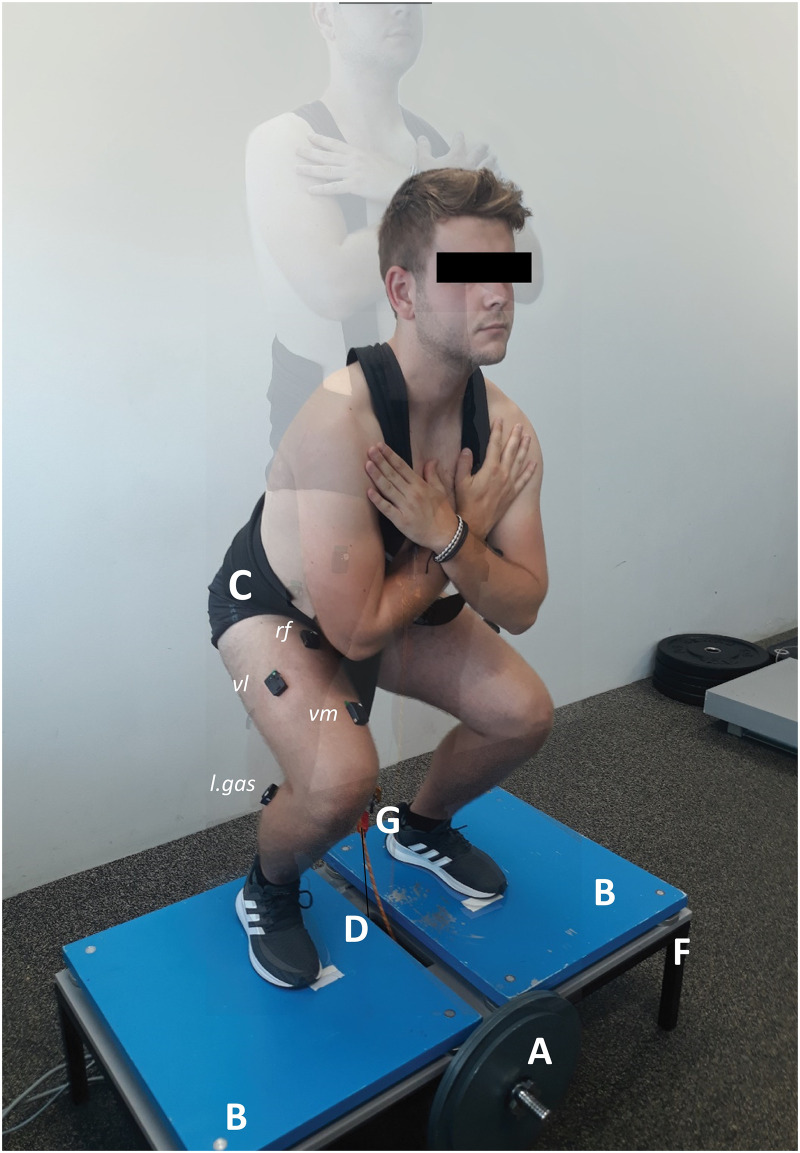
Testing setup. The flywheel (FW) exercise device utilized the inertia of a spinning FW (A) to produce resistance. The FW standing platform (F) with plates (B) size was 1.1 x 0.6 m, rotary shaft diameter was 0.03 m and pulling rope diameter was 0.006 m. A harness (C) was used to aid in performing FW and isometric squats. A draw-wire sensor was installed under the device. The wire originated directly above the center of the axis—to avoid diagonal vertical displacement (D). The distal part of the wire was attached to the harness rope attachment (between legs) (G).

The participants performed squats on a custom-made FW device (Fig 1). Three FW loading conditions were used, i.e. 0.05, 0.125, 0.225 kg∙m^2^. Before each testing session, participants performed a 10-min warm-up as described in detail elswhere [15]. A draw-wire sensor (d = 1250 mm; linearity = ± 0.02%; Way-Con SX-50, Taufkirchen, Deutschland) was fixed perpendicularly to the FW device below the standing surface and a draw-wire was attached to the lifting harness (between legs). The sensor setup provided us with vertical position-time data for the concentric and eccentric phases of the squat. A bilateral force plate system (Type 9260AA, Kistler Instrumente AG, Winterthur, Switzerland) with Kistler MARS software (S2P Ltd., Ljubljana, Slovenia) was used to acquire ground reaction force (*F*) data during maximal voluntary isometric (MVC) contractions. For EMG activity assessment, we used a Trigno Delsys Wireless System (Delsys Inc., Massachusetts, USA), with pre-amplified self-adhesive wireless electrodes (dimensions: 27 x 37 x 15 mm; mass: 14.7 g; electrode material: silver; contact dimension: 5 x 1 mm). After skin preparation (shaving, light abrasion, and cleaning with alcohol; < 5 kΩ), the electrodes were unilaterally placed over soleus (*sol*), *l*.*gas*, semomembranosus (*semi*), biceps femoris (*bf*), *vm*, *vl*, *rf and* glutes maximus (*glut*) muscles according to recommendations for the surface EMG of non-invasive assessment of muscle [16] and secured using flexible adhesive tape (Fig 1). Electrodes were placed on the dominant leg—which was determined as the opposite one to the dominant leg when kicking a ball—in vertical jumping. Ground reaction *F* and vertical position data were simultaneously acquired using a USB Data Acquisition System (synchronized with Delsys Trigger Module and triggered by Kistler MARS software).

Following warm-up, MVC repetitions were performed for the purpose of EMG normalization. Three repetitions (5 s) of maximal isometric exertion against external resistance were performed for each movement: (i) harness squat on FW device in a 90° knee and hip position [17,18] for *vm*, *vl*, *rf*, (ii) good morning deadlift for *semi*, *bf* and *glut*, and (iii) 90° ankle plantar flexion in an upright standing position with fixed pelvis and shoulders for *sol* and *l*.*gas*. Rest periods between repetitions were 60 s and 5 min between the MVC tasks. The participant’s knee and hip angle during normalization was determined with a long arm steel analog goniometer (Saehan Co., Masan, Korea), centered at the lateral epicondyle of the knee or greater trochanter. Loud verbal encouragement by the examiner was provided during all MVC trials.

Thereafter, a total of 15 sets of FW squats were performed. FW loads were applied in counter-balanced random order among the subjects to avoid any systematic inter-load effect. Participants performed 5 sets of 7 repetitions with each of the three loads. The testing protocol was intentionally divided onto sets to reduce the bias of the EMG variables due to fatigue response. The first two repetitions (excluded from data analysis) were intended for FW acceleration and squat amplitude stabilization. The following 5 repetitions were executed with maximal effort and analyzed post-hoc. While the intra-set concentric power output is influenced by the flywheel load used, [19] only 5 repetitions were selected to maintain a high power output—regardless of the load. Participants performed the squat movement from the lower (90° knee angle) position to the full extension of the knees (0° knee angle). Arms were crossed with hands on the opposite shoulders and ankle plantar flexion was not allowed. The participants were instructed to perform the concentric phase as fast as possible while delaying the braking action in the first third of the eccentric phase. Loud verbal encouragement was given to the participants during all testing sessions. To standardize the range of motion, squat amplitude was monitored (real-time feedback from draw-wire sensor on a computer monitor in front of the subject). Moreover, squatting technique (hip and knee flexion angles) was carefully controlled by an experienced researcher. There was 60 s break between sets (same load) and 5 min break between different loads. A numerical rating scale (1–10) [20] in the middle of the rest period was used to record fatigue responses (higher scores indicate more severe fatigue perception).

### Data analysis

**Fig 2:**
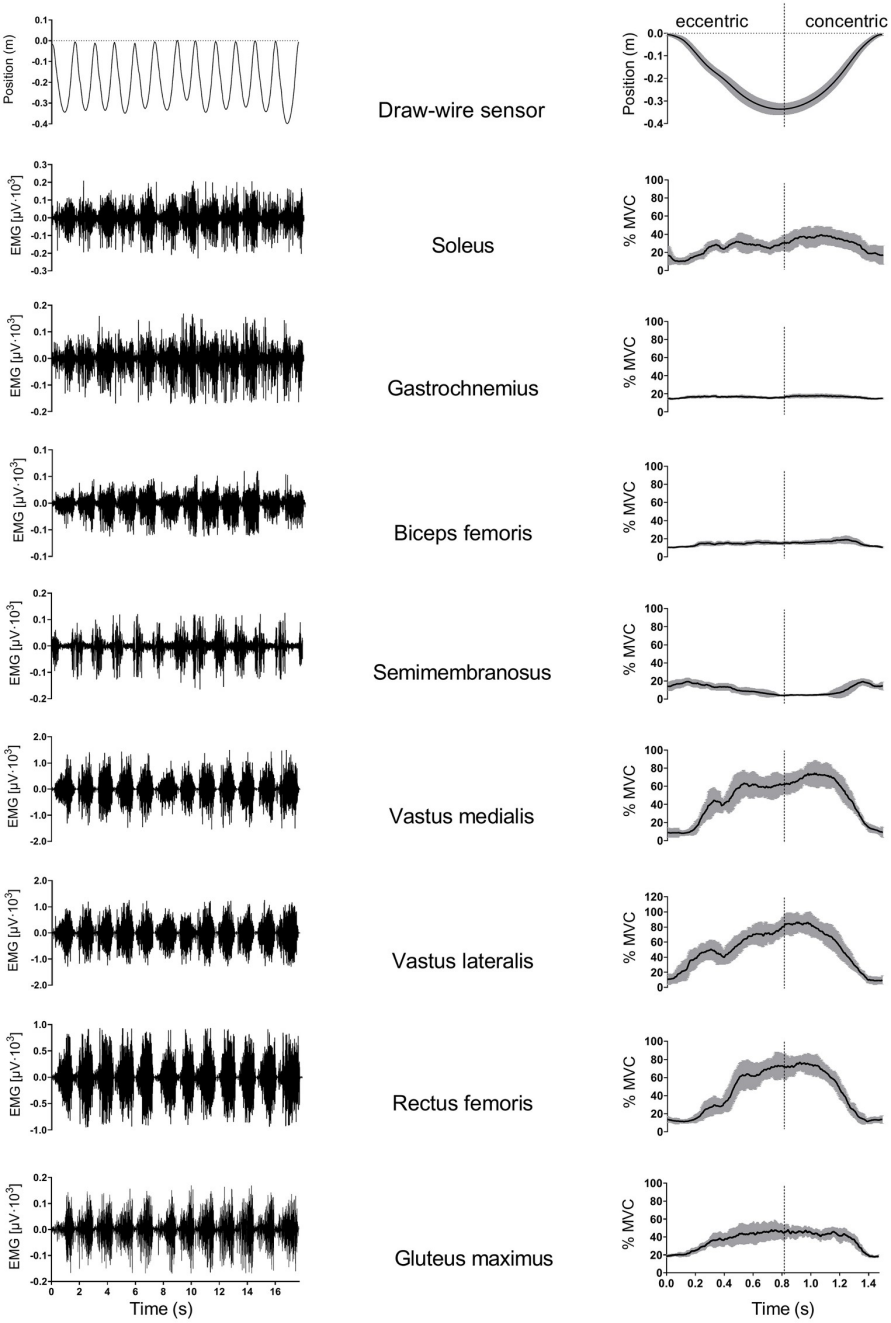
Representation of typical raw and processed vertical position and EMG signal. Data are presented for 12 consecutive squat repetitions at the 0.225 kg∙m^2^ load. The first row represents raw (left) and processed (right) position data. In rows 2–8 raw (left) and processed (right) EMG signals for eight muscles are presented. Repetitions were determined from position data cycles, starting at the highest (approximately 0° knee angle) going through the lowest (approximately 90° knee angle) position and stopping at the highest vertical position. Position data for 12 consecutive repetitions was later time-domain normalized and superimposed (first row, right column). EMG data were firstly filtered and then rectified using root mean square (RMS) function (100 ms) and expressed as a percentage of peak EMG activity during MVC trials (%MVC). Average values (solid line) and standard deviations (grey area) for 12 consecutive time-normalized and superimposed traces are presented in the right column. The concentric area represents the propulsive (concentric) movement and the eccentric area represents braking (eccentric) movement while executing the squat.

Vertical position and EMG activity data were simultaneously collected during FW squats, while ground reaction *F* was collected only during MVC measurements. Data was sampled at a frequency of 1,000 Hz. Position and *F* data were filtered using a moving average filter with 50-ms window, while the EMG data was, firstly, bandpass filtered using Butterworth second-order filter (20–500 Hz) and, secondly, rectified using root mean square (RMS) function (100 ms window length). Raw and processed EMG signals for each representative subject are presented in the Fig 2.

The main outcome variables for the concentric and eccentric phase of each repetition were: (a) peak EMG activity (maximal EMG_RMS_ on the 10% moving window average from position-time data), (b) mean EMG activity (mean EMG_RMS_ from position-time data), and (c) three position-specific variables; mean EMG activity in the first (1./3_mean_), second (2./3_mean_) and third (3./3_mean_) part of the vertical displacement length during the squat derived from the position-time data. The 1./3 corresponds to approximately 9–27°, the 2./3 to 36–54° and the 3./3 to the 63–81° knee flexion angle. Variables were expressed as percentage of peak EMG activity during the MVC trials (%MVC) (calculated as peak value of MVC_RMS_ on a 1 s time window for the peak isometric ground reaction *F* produced).

### Statistical analysis

The obtained averaged outcome variables are reported as means ± standard deviations. Typical error (TE = SD_diff_/√2), coefficient of variation (CV = 100 ∙ (eRMSE/100–1) ≈ 100 ∙ RMSE; RMSE, Square root of the mean square error in the repeated measures ANOVA output) and intraclass correlation coefficient (ICC) were calculated according to [21] and Koo and Li (2016) [22]. ICC values were interpreted according to recent guidelines (< 0.5: poor reliability, 0.5–0.75: moderate reliability, 0.75–0.9: good reliability, and > 0.90): excellent reliability. At the primary level of the analysis, 5 sets of 5 “all out” repetitions were merged and intra-session reliability was calculated between the 25 consecutive repetitions, progressively until all the repetitions were averaged. Values of ICC_2.k_ > 0.95 were considered trustworthy and were included in further analyses. Inter-set reliability was calculated at the secondary level. Twenty-five consecutive repetitions were split into halves and the reliability components (TE, CV, ICC_2.1_ with 95% confidence interval and bias) between the means of the first twelve repetitions in each half were then calculated. The systematic bias between sets was analysed using paired samples t-test. Differences in fatigue scores between loading conditions were tested for statistical significance using one-way repeated measures ANOVA. The assumptions for normality were confirmed using Shapiro-Wilk test and sphericity using Mauchly’s test. Level of significance was set at p < 0.05.

## Results

On average, the fatigue statistics scores significantly increased from 4.48 ± 1.96 after the first loading condition, to 5.04 ± 1.77 after the second and 5.52 ± 1.73 after the third loading condition, F(2, 48) = 6.804, p < 0.05.

**Fig 3:**
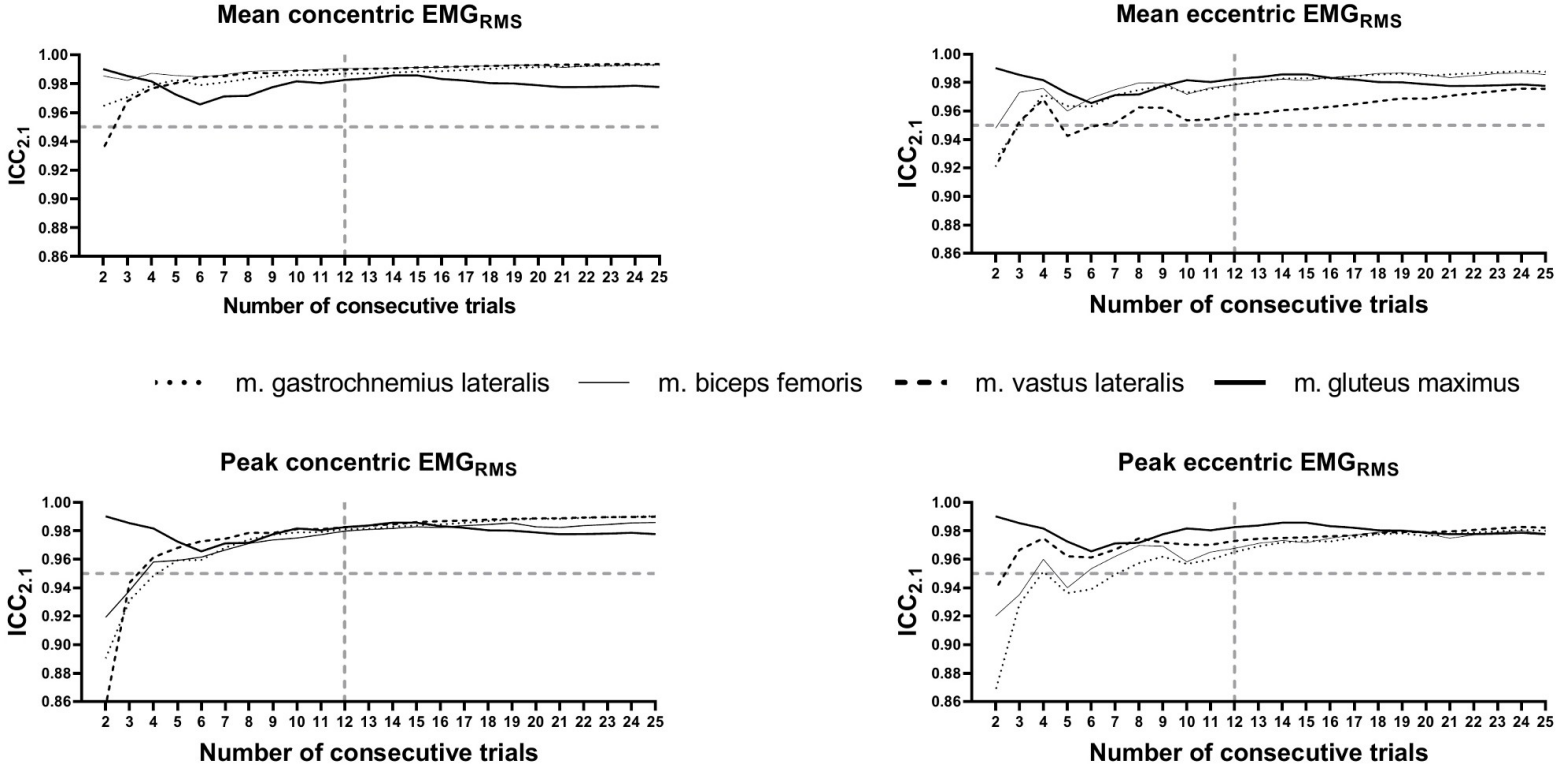
The number of averaged repetitions to assure ICC_2.k_ > 0.95 (dashed horizontal line) for four representative muscles in the concentric and eccentric parts of the squat. The dashed vertical line represents the post-hoc determined cut-off value for the number of consecutively averaged repetitions to meet the reliability criteria for peak and mean EMG_RMS_ outcome variables.

At the primary level, the results showed increasing reliability (ICC_2.k_) with the higher number of averaged repetitions for all EMG_RMS_ variables (Fig 3). Table 2 represents the minimum number of consecutive repetitions to meet the trustworthy criteria. An overall average of 12 consecutive repetitions showed to be the cut-off value for trustworthy (ICC_2.k_ > 0.95) reliability of peak and mean EMG_RMS_ for all muscles in the concentric and eccentric phase of the squat with the exception of the *glut* muscle. Moreover, 89% of position-specific variables (1./3_mean_, 2./3_mean_, 3./3_mean_) meet the trustworthy criteria (ICC_2.k_ > 0.95) when averaging 12 consecutive repetitions. Due to the heterogeneity of the results and total quantity of data, position-specific variables were excluded from further analyses.

**Table 2:** The minimum number of consecutive averaged repetitions to meet the trustworthy criteria (ICC_2.k_ > 0.95).

Load (kg·m^2^)	EMG_RMS_	Concentric								Eccentric							
		sol	l.gas	bf	semi	vm	vl	rf	glut	sol	l.gas	bf	semi	vm	vl	rf	glut
0.05	Peak	3	4	7	2	6	7	3	14	12	6	7	3	11	8	2	19
	Mean	2	2	2	2	3	5	3	16	4	4	2	2	2	2	2	7
	1./3_mean_	13	9	2	10	6	8	4	16	9	9	9	2	8	4	9	6
	2./3_mean_	10	4	11	5	8	8	7	17	17	8	13	7	7	4	3	22
	3./3_mean_	6	6	9	5	11	13	5	12	25	13	2	13	2	8	25	20
0.125	Peak	8	9	8	3	4	5	2	2	6	11	7	3	2	6	4	3
	Mean	7	2	2	3	4	5	4	2	3	7	3	3	2	3	4	2
	1./3_mean_	7	8	3	3	5	7	4	2	6	14	3	4	4	2	25	2
	2./3_mean_	12	9	2	2	8	10	4	2	15	19	3	7	4	4	8	7
	3./3_mean_	11	11	2	5	6	6	5	4	9	12	17	5	3	6	4	22
0.225	Peak	7	5	4	6	3	4	3	2	4	4	4	9	7	3	5	2
	Mean	5	2	2	5	2	3	3	2	4	4	3	3	4	3	8	2
	1./3_mean_	12	5	3	5	5	6	4	2	3	14	3	3	18	22	24	9
	2./3_mean_	11	6	2	2	4	7	6	2	8	7	7	2	9	17	12	12
	3./3_mean_	7	5	2	9	2	3	3	2	11	7	9	3	4	4	7	2

*Note*: Load, Flywheel load; sol, m. soleus; l.gas, m. gastrochnemius lateralis; bf, m. biceps femoris; semi, m. semimembranosus; vm, m. vastus medialis; vl, m. vastus lateralis; rf, m. ractus femoris; glut; m. gluteus maximus; EMG_RMS_, root-mean-square of the EMG signal, ICC_2.k_, Two-way random, average measures, absolute agreement Intra-class correlation coefficient model; concentric, propulsive part of the squat; eccentric, braking part of the squat; 1./3_mean_, 2./3_mean_, 3./3_mean_, mean EMG activity in the first (1./3_mean_), second (2./3_mean_) and third (3./3_mean_) part of vertical displacement length during the squat derived from the position-time data.

Inter-set reliability components from the secondary level of the analysis are presented in Table 3. On average, we found comparable inter-set reliability for peak and mean EMG_RMS_ variables, regardless of the FW load. The muscle activation variables of the eccentric phase of the squat provided us with lower ICC_2.1_ reliability compared to the concentric phase. ICC values ranged from 0.57 (*rf* mean EMG_RMS_ at load 0.05 kg∙m^2^) to 0.99 (*glut* peak EMG_RMS_ at load 0.05 kg∙m^2^) for the concentric phase and from 0.49 (*glut* peak EMG_RMS_ at load 0.225 kg∙m^2^) to 0.96 (*glut* peak EMG_RMS_ at load 0.05 kg∙m^2^) for the eccentric phase related variables. Systematic inter-set bias was found in 23% of the concentric and eccentric phase variables.

**Table 3:** Inter-set reliability of the mean and peak EMG_RMS_ variables.

			Concentric						Eccentric					
muscle	Load (kg·m^2^)	EMG_RMS_	Avg ± SD (%MVC)	Range (%MVC)	TE (%MVC)	CV (%)	ICC_2.1_	t-test sig.	Avg ± SD (%MVC)	Range (%MVC)	TE (%MVC)	CV (%)	ICC_2.1_	t-test sig.
sol	0.05	Mean	41.7 ± 25.4	2.8–92.4	8.9	21.4	0.879	0.420	29.6 ± 16.4	2.1–77.1	6.5	21.8	0.843	0.276
		Peak	57.7 ± 33.9	3.2–133.4	11.2	11.4	0.966	0.072	46.5 ± 26.2	2.5–110.1	10.5	12.9	0.952	0.655
	0.125	Mean	41.1 ± 27.2	2.9–109.3	4.7	21.1	0.861	0.044	38.8 ± 23.7	2.4–87.2	5	17.0	0.908	0.087
		Peak	54.3 ± 34.9	3.5–137.6	8.8	19.3	0.883	0.027	63.5 ± 43.6	2.9–177.7	10.6	22.5	0.844	0.603
	0.225	Mean	33.1 ± 18.4	2.5–74.7	7	16.3	0.926	0.742	40.6 ± 23.5	2.6–86.8	6.9	16.8	0.942	0.102
		Peak	45 ± 23.7	3.4–93.9	10.5	23.3	0.811	0.828	61 ± 38.6	3.2–162.3	10.8	17.7	0.923	0.409
l.gas	0.05	Mean	13.9 ± 12.7	0.6–47.1	2.7	19.7	0.950	0.082	11.9 ± 10.5	0.5–41.5	3.6	30.1	0.889	0.845
		Peak	19.1 ± 16.5	0.8–60.1	4.2	19.6	0.918	0.051	17.7 ± 16.3	0.7–70.8	7.2	14.8	0.943	0.501
	0.125	Mean	12.9 ± 9.4	0.9–43.2	2.5	22.2	0.916	0.044	12.5 ± 9	0.7–37.3	1.9	28.3	0.890	0.003
		Peak	17.9 ± 13	1.2–61.1	5.2	21.8	0.929	0.025	20.7 ± 16.7	0.8–73.1	4.6	40.4	0.812	0.035
	0.225	Mean	12.1 ± 9.1	1.7–36.1	2.7	29.0	0.818	0.633	14 ± 11.8	2–51.9	4	22.0	0.915	0.626
		Peak	17.1 ± 12.9	2–46.7	3.4	20.1	0.928	0.196	21.2 ± 18.7	2.5–86.8	7.1	33.6	0.859	0.840
semi	0.05	Mean	27.6 ± 17.3	4.1–73.1	4.3	15.7	0.939	0.715	27.2 ± 23.2	2.8–134.2	6.5	24.0	0.923	0.694
		Peak	41.9 ± 29.5	4.6–124.8	9.7	15.9	0.941	0.970	42.7 ± 45.5	3.7–294.5	20	15.8	0.950	0.362
	0.125	Mean	25 ± 16.6	4.8–62.4	4	17.0	0.959	0.147	30.3 ± 21.2	6.1–97.1	4.8	14.9	0.944	0.406
		Peak	42.1 ± 34.3	6.7–121.3	8.3	23.1	0.896	0.718	49.5 ± 38.3	7.4–185.4	13.8	46.8	0.807	0.155
	0.225	Mean	23.1 ± 19.6	4.3–112.8	3.9	19.8	0.943	0.289	26.5 ± 16.5	5.2–68.6	3.9	27.8	0.866	0.624
		Peak	37.9 ± 48.9	5.5–315.1	16.7	43.9	0.884	0.300	41.1 ± 28.2	6.9–132.1	7.8	18.9	0.927	0.808
bf	0.05	Mean	21.4 ± 14.5	2.1–74.4	2.6	11.9	0.962	0.013	16.8 ± 10.7	3.1–49.8	3.5	20.6	0.898	0.583
		Peak	33 ± 25.9	3.2–134.6	4.4	16.9	0.923	0.013	29.6 ± 27	4–145.1	7.3	16.0	0.948	0.423
	0.125	Mean	19.5 ± 11.7	2.3–54.8	3.3	14.7	0.945	0.550	23.5 ± 16.6	3.3–79.5	3.8	16.6	0.944	0.234
		Peak	27.3 ± 16.4	3.3–81.5	6.6	13.4	0.964	0.752	40.6 ± 28.7	4.4–140.7	8.9	24.8	0.927	0.302
	0.225	Mean	16.8 ± 10.3	1.5–47.2	2.5	24.3	0.841	0.632	24.1 ± 16.7	1.8–76.3	4	21.8	0.904	0.839
		Peak	23.8 ± 14.6	2.6–55.9	3.6	15.1	0.940	0.259	39.5 ± 28.6	3.4–127.6	9.6	24.3	0.890	0.550
vm	0.05	Mean	53.3 ± 19.4	10.3–88.3	5.3	10.0	0.902	0.004	34.4 ± 13.7	10.9–70.8	4.9	14.2	0.876	0.850
		Peak	68.8 ± 22.6	10.9–106.1	7.1	16.9	0.811	0.051	57.7 ± 20.2	13–99.4	6.6	16.7	0.761	0.471
	0.125	Mean	64.2 ± 24.7	9.3–109.1	10.9	11.7	0.919	0.567	42.3 ± 14.9	12.9–76.2	7.1	10.4	0.844	0.086
		Peak	83.6 ± 32.7	11.4–162.9	14.6	10.3	0.892	0.231	75.6 ± 28.1	15.7–147	13.4	11.4	0.896	0.129
	0.225	Mean	54.3 ± 21.9	11.4–117.4	6.3	17.5	0.796	0.867	49.4 ± 14.6	14.4–88.5	5.1	17.8	0.761	0.006
		Peak	78.9 ± 28.6	14.9–159.8	8.2	10.4	0.918	0.341	77.4 ± 25.7	16.4–148	9.1	11.8	0.868	0.117
vl	0.05	Mean	56.4 ± 24.9	2.5–112.3	10.3	18.3	0.828	0.341	37 ± 19.5	1.6–104.5	5.3	14.2	0.924	0.137
		Peak	77.1 ± 34.1	2.9–157.4	13.6	9.4	0.901	0.684	61.1 ± 27.7	2.4–129.5	7.2	14.9	0.806	0.146
	0.125	Mean	65.3 ± 20.3	17.8–104	6.2	12.7	0.924	0.090	45.3 ± 16.8	19.8–95.5	6.7	16.4	0.757	0.010
		Peak	88 ± 29.1	24.3–153.3	10.2	17.6	0.845	0.063	79 ± 28.7	26.4–158.6	11.6	11.8	0.929	0.010
	0.225	Mean	51.9 ± 23.5	8.6–111.8	6.6	11.6	0.866	0.628	47.3 ± 16.8	8–94.4	7.8	14.7	0.801	0.024
		Peak	79.2 ± 34	11.4–160.4	10.7	13.5	0.884	0.024	75.6 ± 31.1	10.7–168	11.6	15.3	0.850	0.073
rf	0.05	Mean	49.5 ± 25.9	4.1–132	16.3	32.9	0.568	0.033	33.6 ± 22.2	5.1–129.9	14.2	42.4	0.579	0.187
		Peak	71.3 ± 38.1	5.2–184.4	21.9	16.6	0.828	0.055	57 ± 32.8	6.3–186.2	21	26.1	0.613	0.056
	0.125	Mean	61.6 ± 26.6	10–119.6	10.2	15.6	0.895	0.021	32.3 ± 14.5	11.2–72.7	8.4	18.5	0.824	0.014
		Peak	85.8 ± 40.1	17.7–181.6	15.6	30.7	0.645	0.020	65.9 ± 33.7	18.2–183.2	17.1	36.8	0.565	0.017
	0.225	Mean	43.4 ± 20.7	5.4–99	6.8	18.2	0.824	0.572	33.4 ± 15.7	7.9–84.3	6.2	26.0	0.702	0.032
		Peak	69.9 ± 31.5	13.1–145.5	10.1	14.5	0.898	0.446	62.3 ± 27.1	13.6–136.5	9.9	15.9	0.860	0.128
glut	0.05	Mean	58.4 ± 21.3	20.2–101.9	10	17.1	0.782	0.463	34.3 ± 15	10.7–70.7	8	23.5	0.707	0.220
		Peak	90.6 ± 38.1	24–202.3	16.9	15.9	0.990	0.471	65.8 ± 33.9	18.1–181	17.2	12.0	0.961	0.929
	0.125	Mean	63.8 ± 102.3	19.5–585.5	10.2	48.0	0.642	0.266	64.8 ± 43.1	24–252	7.8	39.5	0.537	0.017
		Peak	105.7 ± 173.6	26.2–990.4	17.6	18.7	0.805	0.133	113.2 ± 61.2	40.3–363.8	17.3	26.2	0.748	0.030
	0.225	Mean	39.9 ± 31.7	14.8–233.1	19.1	16.6	0.989	0.668	66.6 ± 39	22.9–303.5	26.3	15.3	0.908	0.183
		Peak	61 ± 47.3	24.5–347.2	27.7	45.3	0.665	0.628	106.4 ± 84.9	36.4–646.9	60.8	57.2	0.485	0.279

*Note*: Load, Flywheel load; *sol*, m. soleus; *l*.*gas*, m. gastrochnemius lateralis; *bf*, m. biceps femoris; *semi*, m. semimembranosus; *vm*, m. vastus medialis; *vl*, m. vastus lateralis; *rf*, m. rectus femoris; *glut*; m. gluteus maximus; EMG_RMS_, root-mean-square of the EMG signal, ICC_2.1_, Two-way random, single measures, absolute agreement Intra-class correlation coefficient model;; concentric, propulsive part of the squat; eccentric, braking part of the squat; 1./3_mean_, 2./3_mean_, 3./3_mean_, mean EMG activity in the first (1./3_mean_), second (2./3_mean_) and third (3./3_mean_) part of vertical displacement length during the squat derived from the position-time data.

## Discussion

The main aim of the study was to define the minimum number of consecutive repetitions that need to be averaged to obtain reliable intra-session EMG variables and, consequently, to asses inter-set reliability of the defined variables. At the primary level of the analysis, we confirmed our first hypothesis with the finding that a minimum of 12 consecutive repetitions should be averaged to obtain trustworthy intra-session EMG outcome variables (ICC > 0.95), excluding position-specific variables due to heterogeneity of the results. Trustworthy intra-session variables provided us with good to excellent inter-set reliability, regardless of muscle, FW load or type of contraction (concentric vs. eccentric). Therefore, we confirmed our secondary level hypothesis. According to the findings, it can be suggested that the minimum number of repetitions that should be averaged in one set is 12 to ensure trustworthy intra-session reliability of the peak and mean EMG variables. To ensure that influence of fatigue is excluded from the testing results, we suggest performing two sets of six repetitions at a certain load to achieve the suggested number of intra-set repetitions.

In the FW resistance exercise, *P* and *F* vary depending on the tempo of execution, which may highlight the imprecision of prescribing FW loading and reflect the lack of reliability in performance testing. We observed that 12 consecutively averaged repetitions represented the cut-off value that ensures trustworthy reliability of the EMG variables among all three FW loads used, when excluding position-specific variables and *glut* muscle from the first phase of the analysis. A conclusion of trustworthiness (ICC_2.k_ > 0.95) was made due to the possible influence of inter-individual variability on the magnitude of ICCs [23]. Due to the high heterogeneity of subjects (high CV), a large ICC can be obtained even when consistency is poor [24]. Moreover, when analysing specific muscles (e.g. only *vl*), less than 12 repetitions are adequate to meet the trustworthy intra-session criteria—with the help of the Table 3. Position-specific variables showed lower reliability when averaging several consecutive repetitions and higher result variations. When processing position-specific EMG signals—in respect of different muscles—from 2 to 25 repetitions should be averaged and, consequently, the results should be interpreted with caution.

The main advantage of our study is the quantity of valuable data collected using valid modern technology, i.e. force plates, draw-wire linear positional sensor and 8-channel wireless EMG system. Moreover, direct transfer rope-FW offers basic FW resistance exercise conditions, enabling easily controllable exercise intensities. Although we used a custom-made FW device with three FW loading conditions, we do not see a functional divergence to the commercially available devices that are frequently used for this sort of training. The results of our study are reproducible for simultaneous measurements of vertical displacement and muscles EMG activity. Some commercially available devices enable calculation of mechanical variables (i.e. vertical displacement) from axis rotation data alone. In such cases, researchers should be cautious about the following characteristics of the FW devices, as they can affect the fundamental metric characteristics: strap/rope winding around the axis, direct/pulley mechanism rope to axis transfer and cylinder/cone shaped axis. In terms of fatigue rating, although the scores increased from the first to the last FW load, fatigue influence should be equally distributed between different loads as these were executed in a different random order for each participant.

There were several limitations with the testing procedure that should be noted. At the transition from the eccentric to the concentric phase of the squat, we observed a certain decrease in the participant’s balance and therefore inter-participant variability. Unsteadiness can potentially affect squatting performance, especially using high FW loads, although we have done our best to ensure maximum squat execution among all FW loads. On some occasions, FW harness discomfort could also have influenced squatting performance. Sabido et al (2018) [19] emphasised the importance of the familiarization process, showing that the participants’ experience plays an important role in some variables, such as peak *P* output and eccentric overload. As yet, we lack information about EMG variables concerning the familiarization process. Familiarization in our study was shorter than suggested [19]. Nevertheless, we found good to excellent inter-set reliability using each of the three FW loads. We believe that the consistency of the muscle activation results reflects the highly-strength-trained participants and of the equipment. The direct transfer rope-FW shaft used offers better, more fluent movement feeling, and consequently better squat depth control. Based on these findings, stabilization, comfort requirements, familiarization procedures and consequently inter-visit reliability should be taken into account and explored further.

In the present study, we only concentrated on the inter-set reliability of the peak and mean EMG_RMS_ variables due to the large dataset involved. It should be noted that the main findings of the study are also applicable when analysing position-specific variables, especially when exploring the neuromechanical principles responsible for adaptations in FW resistance training. It has been found that training adaptations relating to the depth of a squat differently influences adaptations in strength, sprinting and jumping abilities [25].

Similar to pedalling motion [26], we found that consecutive FW squat repetitions result in onsets and offsets of the main burst of EMG activity. We believe such bursts are consequences of mechanical restraints of FW loading conditions and are therefore vertical displacement dependent. In future research, the range of the active phase should be defined (duration between the onset and the offset of the muscle activity), which should also positively influence result reliability, especially with respect to position-specific results. In addition, we suggest analysing the EMG amplitude to *F* ratio while following specific training adaptations [27]. With additional research, it is possible that the linear slope coefficient of the EMG amplitude to the squat vertical ground reaction *F* spectrum may be useful for examining neural vs. hypertrophic adaptations to strength training [28] in a specific—i.e. FW—conditions.

By using reliability data as the decision-making criteria in this process, the testing protocol has likely been optimised. The results should contribute to the optimization of EMG measurements using FW squat devices and therefore help research practitioners to obtain confident results. According to the findings, it can be suggested that the minimum number of repetitions that should be averaged to ensure trustworthy intra-session reliability of EMG variables is 12. Moreover, our data demonstrates that 12 consecutive averaged squat repetitions in a single set achieves good to excellent inter-set reliability of the EMG variables. The results are expected to lead the standardization of a methodology for quick and less prone to fatigue assessing EMG activity of leg muscles using FW squats. Taking these results into account, activation of leg muscles can be confidently studied in intra-session repeated-measures study designs. In addition, researchers should be aware of their FW device’s characteristics to obtain the most relevant EMG results.

## References

[1] Norrbrand L, Pozzo M, Tesch PA. Flywheel resistance training calls for greater eccentric muscle activation than weight training. Eur J Appl Physiol. 2010;110: 997–1005. 10.1007/s00421-010-1575-7 20676897

[2] Onambele GL, Maganaris CN, Mian OS, Tam E, Rejc E, McEwan IM, et al Neuromuscular and balance responses to flywheel inertial versus weight training in older persons. J Biomech. 2008;41: 3133–3138. 10.1016/j.jbiomech.2008.09.004 18976996

[3] Filho MB, Manso JG, Sarmiento S, Medina G. Hamstrings Co-Contraction In Knee Extension During Isoinertial Strength Work. Rev Bras Biomecânica. 2008;9: 12–17.

[4] Carroll KM, Wagle JP, Sato K, Christopher B. Taber NY, Bingham GE, et al Characterising overload in inertial flywheel devices for use in exercise training. Sport Biomech. 2018;18: 1–12. 10.1080/14763141.2018.1433715 29558854

[5] Oliveira AS, Gizzi L, Farina D, Kersting UG. Motor modules of human locomotion: Influence of EMG averaging, concatenation, and number of step cycles. Front Hum Neurosci. 2014;8: 1–9. 24904375 10.3389/fnhum.2014.00335 PMC4033063

[6] Pozzo M, Alkner B, Norrbrand L, Farina D, Tesch PA. Muscle-fiber conduction velocity during concentric and eccentric actions on a flywheel exercise device. Muscle and Nerve. 2006;34: 169–177. 10.1002/mus.20574 16688721

[7] Naczk M, Naczk A, Brzenczek-Owczarzak W, Arlet J, Adach Z. Impact Of Inertial Training On Strength And Power Performance In Young Active Men. J Strength Cond Res. 2016;30: 1534–1539. 27457914 10.1097/JSC.0000000000000217

[8] Norrbrand L, Tous-Fajardo J, Vargas R, Tesch P. Quadriceps muscle use in the flywheel and barbell squat. Aviat Sp Environ Med. 2011;82: 13–19. 10.3357/asem.2867.2011 21235100

[9] Alkner BA, Bring DKI. Muscle Activation During Gravity-Independent Resistance Exercise Compared to Common Exercises. Aerosp Med Hum Perform. 2019;90: 506–512. 10.3357/AMHP.5097.2019 31101135

[10] Alkner BA, Tesch PA. Efficacy of a gravity-independent resistance exercise device as a countermeasure to muscle atrophy during 29-day bed rest. Acta Physiol Scand. 2004;181: 345–357. 10.1111/j.1365-201X.2004.01293.x 15196095

[11] Duchateau J, Baudry S. Insights into the neural control of eccentric contractions. J Appl Physiol. 2013;116: 1418–1425. 10.1152/japplphysiol.00002.2013 23429873

[12] Herzog W. Why are muscles strong, and why do they require little energy in eccentric action? J Sport Heal Sci. 2018;7: 255–264. 10.1016/j.jshs.2018.05.005 30356622 PMC6189244

[13] Maffiuletti NA, Aagaard P, Blazevich AJ, Folland J, Tillin N, Duchateau J. Rate of force development: physiological and methodological considerations. Eur J Appl Physiol. 2016;116: 109–1116. 10.1007/s00421-016-3346-6 26941023 PMC4875063

[14] Reaz MBI, Hussain MS, Mohd-Yasin F. Techniques of EMG signal analysis: Detection, processing, classification and applications. Biol Proced Online. 2006;8: 11–35. 10.1251/bpo115 16799694 PMC1455479

[15] Spudić D, Smajla D, Šarabon N. Validity and reliability of force—velocity outcome parameters in flywheel squats. J Biomech. 2020;107: 109824 10.1016/j.jbiomech.2020.109824 32517866

[16] Hermens HJ, Freriks B, Disselhorst-Klug C, Rau G. Development of recommendations for SEMG sensors and sensor placement procedures. J Electromyogr Kinesiol. 2000;10: 361–374. 10.1016/s1050-6411(00)00027-4 11018445

[17] Trindade TB, De Medeiros JA, Dantas PMS, De Oliveira Neto L, Schwade D, De Brito Vieira WH, et al A comparison of muscle electromyographic activity during different angles of the back and front squat. Isokinet Exerc Sci. 2020;28: 1–8. 10.3233/IES-193142

[18] Marchetti PH, Jarbas da Silva J, Schoenfeld BJ, Nardi PSM, Pecoraro SL, D’Andréa Greve JM, et al Muscle Activation Differs between Three Different Knee Joint-Angle Positions during a Maximal Isometric Back Squat Exercise. J Sports Med. 2016; 1–6. 10.1155/2016/3846123 27504484 PMC4967668

[19] Sabido R, Hernández-Davó JL, Pereyra-Gerber G. Influence of Different Inertial Loads on Basic Training Variables During the Flywheel Squat Exercise. Int J Sports Physiol Perform. 2018;13: 482–489. 10.1123/ijspp.2017-0282 28872379

[20] Micklewright D, St Clair Gibson A, Gladwell V, Al Salman A. Development and Validity of the Rating-of-Fatigue Scale. Sport Med. 2017;47: 2375–2393. 10.1007/s40279-017-0711-5 28283993 PMC5633636

[21] Hopkins WG. Measures of Reliability in Sports Medicine and Science. Sport Med. 2000;30: 1–15. 10.2165/00007256-200030010-00001 10907753

[22] Koo TK, Li MY. A Guideline of Selecting and Reporting Intraclass Correlation Coefficients for Reliability Research. J Chiropr Med. 2016;15: 155–163. 10.1016/j.jcm.2016.02.012 27330520 PMC4913118

[23] Atkinson G, Nevill AM. Statistical methods for assessing measurement error (reliability) in variables relevant to sports medicine. Sport Med. 1998;26: 217–238. 10.2165/00007256-199826040-00002 9820922

[24] Weir J. Quantifying Test-Retest Reliability Using The Intraclass Correlation Coefficient And The Sem. J Strength Cond Res. 2005;19: 231–240. 10.1519/15184.1 15705040

[25] Rhea MR, Kenn JG, Peterson MD, Massey D, Simão R, Marin PJ, et al Joint-Angle Specific Strength Adaptations Influence Improvements in Power in Highly Trained Athletes. Hum Mov. 2016;17: 43–49. 10.1515/humo-2016-0006

[26] Fonda B, Panjan A, Markovic G, Sarabon N. Adjusted saddle position counteracts the modified muscle activation patterns during uphill cycling. J Electromyogr Kinesiol. 2011;21: 854–860. 10.1016/j.jelekin.2011.05.010 21684759

[27] Moritani T, DeVries H. Neural Factors Versus Hypertrophy. Am J Phys Med. 1979;58: 115–130. 453338

[28] Michael L, Stock M, Chappell A. Electromyographic Amplitude vs. Concentric and Eccentric Squat Force Relationships for Monoarticular and Biarticular Thigh Muscles. J Strength Cond Res. 2014;28: 328–338. 10.1519/JSC.0b013e3182a1f434 23897014

